# Editorial: Pathogenomics of the genus *Brucella* and beyond, volume II

**DOI:** 10.3389/fmicb.2024.1370330

**Published:** 2024-01-25

**Authors:** Axel Cloeckaert, R. Martin Roop, Holger C. Scholz, Adrian M. Whatmore, Michel S. Zygmunt

**Affiliations:** ^1^INRAE, Université de Tours, UMR, ISP, Nouzilly, France; ^2^Department of Microbiology and Immunology, Brody School of Medicine, East Carolina University, Greenville, NC, United States; ^3^Centre for Biological Threats and Special Pathogens, Highly Pathogenic Microorganisms (ZBS 2), Robert Koch Institute, Berlin, Germany; ^4^Department of Bacteriology, Animal and Plant Health Agency, Weybridge, United Kingdom

**Keywords:** *Brucellaceae*, *Brucella*, *Ochrobactrum*, genetics/genomics, diversity, evolution, cell envelope, virulence

*Brucellae* are Gram-negative, facultative, intracellular bacteria that can infect humans and many species of animals. Brucellosis is an economically important disease in production animals worldwide causing abortion and infertility. Human brucellosis has usually been associated with an animal reservoir of *Brucella* spp. and transmission occurs mainly via the food chain, in particular through dairy products, or via direct contact with diseased animals. The genus *Brucella* has historically been classified into six species, according to their preferential animal host, of which the most pathogenic for humans are *B. melitensis, B. suis*, and *B. abortus*. The genus *Brucella* has been further expanded with a set of new species discovered from the 1990's mainly from wildlife, including marine mammal and amphibian species. Comparative genomics has provided insight into the evolutionary history of species belonging to the genus *Brucella* but has not yet facilitated identification of the underlying mechanisms involved in host preference or diseases caused in their respective hosts.

Following up on the success of the Research Topic *Pathogenomics of the genus Brucella and beyond* we have launched a volume II of the Research Topic. The current Research Topic consisted of 11 Original Research articles and one Opinion article.

Three articles focused on diversity, molecular epidemiology, and evolutionary history of *B. abortus*. The first characterized Ethiopian *B. abortus* isolates from an outbreak in cattle. By comparative genomic analysis with other *B. abortus* sequences available in public databases, Edao et al. reported that the Ethiopian isolates formed a unique cluster within the *B. abortus* phylogeny closely related to a small number of isolates previously described in sub-Saharan Africa, and further analysis suggested a potential evolutionary origin for the *B. abortus* species in East Africa. The second *B. abortus* study by Shevtsov et al. reported the population structure of *B. abortus* in Kazakhstan and its comparison with worldwide genetic diversity of this species. A Bayesian phylodynamic approach suggested that *B. abortus* lineages currently circulating in Kazakhstan were introduced in the nineteenth–twentieth centuries from Europe, mainly from Russia (North Caucasia). The topology of the observed comparative phylogeny of *B. abortus* genome sequences combined with human history pointed also to East Africa as the current most parsimonious scenario for the origin of *B. abortus*. Finally, Janke et al., in a more comprehensive study, reported the global phylogenomic diversity of *B. abortus*. The authors confirmed 4 major *B. abortus* clades, named A to D. Two of these clades, clade A (median estimate date 972 CE; range 781–1,142 CE) and clade B (median date 150 BCE; range 515 BCE−164 CE), were exceptionally diverse for this species and are exclusively of African origin. The third clade, clade C (median date 949 CE; range 766–1,102 CE), had most isolates coming from a broad swath of the Middle East, Europe, and Asia, also had relatively high diversity. Finally, the fourth and most recent major clade, clade D (median date 1,467 CE; range 1,367–1,553 CE) comprises the large majority of genomes in a dominant but relatively monomorphic group that predominantly infects cattle in Europe and the Americas.

In addition to the above *B. abortus* studies, Xue et al. characterized native circulating *B. melitensis* lineages causing a brucellosis epidemic in Qinghai, China. A global-scale phylogenetic analysis indicated that 54 strains, mostly human, sorted into six subclades, four of which formed independent lineages, suggesting that the increase in the incidence rate of human brucellosis may be caused by local circulating lineages.

The *Brucella* genus also comprises novel species isolated from amphibians. Scholz et al. reported for the first time the isolation of *B. inopinata* from a White's tree frog (*Litoria caerulea*). The species *B. inopinata* was initially reported from a human case and its animal or environmental origin, or any other contaminating source, was not yet identified. Genomic analyses unequivocally classified the exotic frog isolate as belonging to *B. inopinata*. The isolation of *B. inopinata* from a frog, along with other reports of human infection by atypical *Brucella*, raises further the question of whether atypical *Brucella* could pose a risk to human health.

Four articles dealt with pathogenic or immune mechanisms involved in *B. abortus* infection. The first Opinion article by Oliveira and Guimarães raised the question on how the crosstalk between innate immune sensors and metabolic pathways can affect the outcome of *B. abortus* infection. The authors discussed recent developments in the metabolic reprogramming of macrophages and speculate on the prospect of targeting immunometabolism in an effort to develop novel therapeutics to treat *Brucella* and other bacterial infections. A second Original Research article by Muruaga et al. reported the biochemical and functional characterization of *B. abortus* cyclophilins. Cyclophilins of *Brucella* (CypA and CypB) are enzymes encoded by genes that are upregulated within the intraphagosomal replicative niche and required for stress adaptation and host intracellular survival and virulence. In the present study, the authors characterized these cyclophilins from a biochemical standpoint by studying their PPIase activity, chaperone activity, and oligomer formation. In summary, according to the authors *Brucella* cyclophilins come in two different “flavors:” eukaryotic and prokaryotic. CypA and CypB differ in various immunological and biochemical properties, despite their high degree of sequence similarity and conserved functional features. The importance of dimer formation and PPIase activity of CypB for a progressive infection were highlighted in an animal model. These findings shed some light on the potential novel functions of *Brucella* Cyps, some of them could be due to the putative role of CypB as an effector bacterial protein. Regarding innate immunity mechanisms, Santos et al. reported that caspase-8 but not caspase-7 influences inflammasome activation acting in the control of *B. abortus* infection. Programmed cell death (PCD) is an important mechanism of innate immunity against bacterial pathogens. The innate immune PCD pathway involves the molecules caspase-7 and caspase-8, among others. By several *in vivo* experimental approaches, the authors showed the important role of caspase-8 in inflammasome activation and innate immunity against *B. abortus* infection. Finally, regarding *B. abortus* virulence regulation, Castillo-Zeledón et al. showed that the two-component system response regulator BvrR binds to three DNA regulatory boxes in the upstream region of *omp25* encoding a major outer membrane protein. The two-component regulatory system BvrR/BvrS modulates the expression of genes required to transition from extracellular to intracellular lifestyles. Among the genes regulated, *omp25* is positively regulated by this regulatory system. The authors propose that BvrR binds directly to up to three regulatory boxes and probably interacts with other transcription factors to regulate *omp25* expression.

Three articles of the Research Topic focused on means to combat brucellosis, with the development of improved vaccines and of *in vivo* experimental infection models. Mena-Bueno et al. developed and characterized a *wzm*-mutant *B. melitensis* Rev1 vaccine candidate, altered in its cell envelope, and showing improved vaccine properties in a mouse model of infection. The lipopolysaccharide (LPS) O-polysaccharide (O-PS) is a major virulence factor in *Brucella*. After synthesis in the cytoplasmic membrane, O-PS is exported to the periplasm by the Wzm/Wzt system, where it is assembled into a LPS. The *wzm* gene deletion in the Rev1 vaccine strain resulted in lack of external O-PS production as expected, but in addition triggered changes in genetic transcription and in phenotypic properties associated with the outer membrane and cell wall. This highly attenuated mutant strain proved to excel also as an immunogenic and effective vaccine against *B. melitensis* and *B. ovis* in mice, revealing that low persistence is not at odds with efficacy. Overall, these attributes, and the minimal serological interference induced in sheep, make Rev1Δ*wzm* a highly promising vaccine candidate. Nandini et al. performed immuno-profiling of *Brucella* proteins for developing improved vaccines and DIVA (Differentiating Infected from Vaccinated Animals) capable serodiagnostic assays for brucellosis. Several immunodominant proteins were identified in this study by high throughput immunoprofiling of *B. melitensis* protein microarray using brucellosis-positive human and animal serum samples. Among the seroreactive proteins, the Dps protein, strongly reacted with brucellosis-positive serum samples, but it did not react with sera from *B. abortus* S19-vaccinated cattle, indicating DIVA capability. A prototype lateral flow assay and indirect ELISA based on Dps protein exhibited high sensitivity, specificity, and DIVA capability. Finally, Hensel et al. assessed the guinea pig model of infection in comparison to the mouse model of infection, together with intratracheal inoculation, as a model for male reproductive brucellosis. Strains tested were the *B. melitensis* 16M virulent strain and the derived vaccine candidate 16 MΔ*vjbR*. Due to the ability to evaluate for both colonization and inflammation, the authors concluded that guinea pigs seemed the better model relative to the mouse model, not only for assessing host-pathogen interactions but also for future vaccine development efforts.

In summary, the current volume II Research Topic on *Brucella* pathogenomics, contributed to increase our knowledge in (i) the global spread and evolutionary history of pathogenic *Brucella* species, (ii) the pathogenic or immune mechanisms involved in *B. abortus* infection, and (iii) novel approaches and vaccine candidates to combat brucellosis.

## Author contributions

AC: Writing – original draft, Writing – review & editing. RR: Writing – original draft, Writing – review & editing. HS: Writing – original draft, Writing – review & editing. AW: Writing – original draft, Writing – review & editing. MZ: Writing – original draft, Writing – review & editing.

